# Monocyte Locomotion Inhibitory Factor Produced by *E. histolytica* Improves Motor Recovery and Develops Neuroprotection after Traumatic Injury to the Spinal Cord

**DOI:** 10.1155/2013/340727

**Published:** 2013-11-03

**Authors:** Gabriela Bermeo, Antonio Ibarra, Elisa García, Adrian Flores-Romero, Guadalupe Rico-Rosillo, Rubén Marroquín, Humberto Mestre, Carmina Flores, Francisco Blanco-Favela, Raúl Silva-García

**Affiliations:** ^1^Unidad de Investigación Médica en Inmunología, Hospital de Pediatría, CMN-Siglo XXI, IMSS, Avenida Cuauhtémoc 330, Col. Doctores, Delegación Cuauhtémoc, 06720 México, DF, Mexico; ^2^Posgrado en Ciencias en Inmunología, Instituto Politécnico Nacional, Prolongación de Carpio y Plan de Ayala, Col. Santo Tomas, 11340 México, DF, Mexico; ^3^Facultad de Ciencias de la Salud, Universidad Anáhuac, México Norte, Avenida Universidad Anáhuac 46, Col. Lomas Anáhuac, 52786 Huixquilucan, MEX, Mexico; ^4^Centro de Investigación del Proyecto CAMINA, A.C, Tlalpan 4430, Col. Toriello Guerra, 14050 México, DF, Mexico; ^5^División de Investigación, Facultad de Medicina, UNAM, Avenida Universidad 3000, 04510 México, DF, Mexico; ^6^Facultad de Estudios Superiores Zaragoza, UNAM, Batalla 5 de Mayo/Fuerte de Loreto, Col. Ejercito de Oriente, 09230 México, DF, Mexico

## Abstract

Monocyte locomotion inhibitory factor (MLIF) is a pentapeptide produced by *Entamoeba histolytica* that has a potent anti-inflammatory effect. Either MLIF or phosphate buffered saline (PBS) was administered directly onto the spinal cord (SC) immediately after injury. Motor recovery was evaluated. We also analyzed neuroprotection by quantifying the number of surviving ventral horn motor neurons and the persistence of rubrospinal tract neurons. To evaluate the mechanism through which MLIF improved the outcome of SC injury, we quantified the expression of inducible nitric oxide synthase (iNOS), interleukin-10 (IL-10), and transforming growth factor-**β** (TGF-**β**) genes at the site of injury. Finally, the levels of nitric oxide and of lipid peroxidation were also determined in peripheral blood. Results showed that MLIF improved the rate of motor recovery and this correlated with an increased survival of ventral horn and rubrospinal neurons. These beneficial effects were in turn associated with a reduction in iNOS gene products and a significant upregulation of IL-10 and TGF-**β** expression. In the same way, MLIF reduced the concentration of nitric oxide and the levels of lipid peroxidation in systemic circulation. The present results demonstrate for the first time the neuroprotective effects endowed by MLIF after SC injury.

## 1. Introduction

 Spinal cord (SC) injury causes structural alterations that result in permanent sequels to the nervous system. These are the result of a series of pathological events that impede the normal electric conductivity of the SC rendering the functional relay of information past the level of injury impossible [1, 2]. Once the mechanical injury of the SC is produced (primary injury), there is vessel rupture and neural tissue disruption. Immediately after the initial phase of injury, a series of autodestructive mechanisms are triggered (secondary injury), causing more damage to SC parenchyma and, thus, a chronic neurodegenerative process [2]. The injury causes neurogenic shock and together with the vascular damage, hemorrhage, and ischemia of the SC, there is a state of systemic inflammation. The excessive release of vasoactive mediators by the injured nervous system induces lipid peroxidation, increases intracellular calcium levels, triggers excitotoxicity, and installs several other harmful events [3, 4].

Free radicals such as superoxide anion (O_2_^∙−^), hydroxyl (OH^−^), nitric oxide (NO), and peroxynitrite (ONOO^−^, produced by the reaction between O_2_^∙−^ and NO radicals) are powerful nitrates and oxidizing agents [1, 5–9]. The excessive production of these compounds is associated with neurotoxicity and further contributes to the secondary injury. Lipid peroxidation is the main mechanism by which free radicals contribute to promote damage in the central nervous system (CNS) [6–8, 10]. SC injury elicits an intense inflammatory response of neutrophils, mast cells, and a large number of macrophages infiltrating the site of injury. Activated neutrophils and macrophages produce O_2_^∙−^ and NO (also generated by platelets, endothelial cells, and microglia) and contribute to lipid peroxidation. This cellular infiltrate is associated with the impairment and the amount of tissue damage after the injury, as well as with the gradual degeneration of vascular and neural tissues [9, 10]. This is why several therapeutic strategies are being developed to protect the SC against these phenomena. Some of these therapies are based on the modulation of the inflammatory response helping to avoid the progress of immune cell-mediated lipid peroxidation [11–14]. In our laboratory, we isolated, purified, and sequenced a pentapeptide (Met-Gln-Cys-Asn-Ser) produced by *Entamoeba histolytica* (*E. histolytica*), which is called monocyte locomotion inhibitory factor (MLIF). MLIF is synthesized as a protein or as a large peptide and then is cleaved by a protease to become active [15]. This peptide is capable of diminishing the locomotion of mononuclear phagocytes in vitro [16]. Studies in our laboratory have demonstrated that MLIF reduces the ability of monocytes and neutrophils of generating reactive oxygen intermediates (ROI), therefore reducing NO production and the concentration of cyclic guanine monophosphate (cGMP) [17–19]. Aside from this, MLIF enhances the prevalence of a Th2 phenotype, which is critical for regulating proinflammatory responses [20, 21]. 

MLIF has been broadly investigated and at the moment, there is also scientific evidence on the effect of this molecule upon the expression of genes involved in angiogenesis, extracellular matrix synthesis/degradation, axonal guidance, and even those encoding for certain growth factors [20]. In vivo, MLIF delays the arrival of mononuclear cells in Rebuck human skin windows test, counteracts the formation of pericardial adherences, and inhibits skin retarded hypersensitivity against dinitrochlorobenzene (DNCB) and the expression of adherence molecules such as integrin alpha 4 beta 1 (VLA-4) and vascular cell adhesion molecule-1 (VCAM-1) [22, 23]. MLIF's inherent biological effects make it a candidate to be used in inflammatory and neurodegenerative processes, especially those with intense free radical production, such as SC injury. The use of MLIF in SC injury could reduce the damage inflicted by the secondary injury by diminishing the inflammatory response and free radicals release.

In this work, we studied the effect of MLIF on experimental SC injury. For this purpose, we evaluated the motor recovery, survival of ventral horn and rubrospinal neurons, NO production (nitrites), lipid peroxidation, and the expression of some genes related to inflammation [inducible nitric oxide synthase (iNOS), interleukin-10 (IL-10), and transforming growth factor-*β* (TGF-*β*)]. 

We found that MLIF decreased NO production, lipid peroxidation, and iNOS expression. It also increased the survival of ventral horn and rubrospinal neurons and these findings correlated with a significant recovery in motor coordination. The neuroprotective effects exerted by MLIF could be induced by the increased expression of IL-10 and TGF-*β*. The present results demonstrate for the first time the neuroprotective effects endowed by MLIF after SC injury.

## 2. Materials and Methods 

### 2.1. Experimental Design

This investigation was comprised of two phases. The first phase evaluated the motor recovery, the survival of ventral horn (in spinal cord) and rubrospinal neurons (in the red nucleus) in three groups of rats (10 animals per group): (1) Sham-operated (Sham), (2) SC injured + MLIF, and (3) SC injured + phosphate buffered saline (PBS). In the second phase, we evaluated the effect of MLIF on plasmatic lipid peroxidation and nitric oxide levels and the intraspinal expression of iNOS, IL-10, and TGF-*β* in three groups of rats (12 animals per group): (1) Sham-operated (Sham), (2) SC injured + MLIF, and (3) SC injured + PBS. Animals were sacrificed at 3 hours (*n* = 4), 7 days (*n* = 4), and 14 days (*n* = 4) after injury. 

### 2.2. Monocyte Locomotion Inhibitory Factor (MLIF)

MLIF was commercially acquired from the American Peptide Company Co. (Sunnyvale, CA, USA) with purity above 95%; it was diluted in PBS pH 7.4 to a final concentration of 4 *μ*g/*μ*L. A Limulus assay (Amoebocyte Lysate Endosafe KTA Charles River Endosafe INC, Charleston, SC, USA) was performed to ensure that the preparations were endotoxin-free (LPS < 0.3 pg) prior to storage at −70°C. 

### 2.3. Animals and Ethics Statement

Ninety-six female Sprague-Dawley rats (13-14 weeks old and 200–250 g of body weight) were obtained from Proyecto Camina A.C. (México, D.F.). All procedures were in accordance with the National Institutes of Health (US) Guide for the Care and Use of Laboratory Animals and the Mexican official Norm for production, care, and usage of laboratory animals (NOM-062-Z00-1999). All animal procedures were approved by the National Commission for Scientific Investigation and Animal Bioethics and Welfare Committee of the Instituto Mexicano del Seguro Social (ID: 2008-785-064) and the National Council of Science and Technology of Mexico (CONACyT) (ID: 168202).

### 2.4. Spinal Cord Injury and MLIF/PBS Administration

Rats were subjected to a SC contusion as previously described [24]. Thirty minutes after an intramuscular injection of ketamine (50 mg/kg, Probiomed, Mexico City, Mexico) and xylazine (10 mg/kg, Fort Dodge Laboratories, Fort Dodge, Iowa), a 10 g. rod was dropped onto the spinal cord from a height of 25 mm. using the NYU impactor (NYU, New York). This device has shown to inflict a well-calibrated contusive injury of the SC. Surgical access to the SC was achieved with a laminectomy of the T9 vertebral body; the contusive injury was inflicted at this level as well. 

Once the lesion was inflicted, 50 *μ*L of MLIF or PBS were directly irrigated at the site of injury. After MLIF/PBS administration, the animals were sutured with absorbable dexon and nylon thread. Animals were kept in a room with controlled temperature and humidity.

### 2.5. Animal Care

Injured rats were kept on sterilized sawdust and were provided with filtered water, both of which were changed every day. Their bladder was evacuated by means of an abdominal massage performed 2-3 times a day until normal urination was regained. All rats were carefully monitored to avoid and detect urinary tract infections or any other sign of systemic infection. The animals were treated with 64 mg/kg of enrofloxacin per day when hematuria was presented during the first 10 days after the injury was inflicted.

### 2.6. Assessment of Motor Recovery

The Basso, Beattie and Bresnahan locomotor ability open-field test [25] was used to evaluate the rats' motor ability every 7 days, for 8 weeks (56 days) after injury. Recovery was rated on a scale from 0 (total paralysis) to 21 (complete motility). During double-blind evaluation, the animals were placed on an open-field observation surface and were allowed to walk freely for 5–7 minutes. 

### 2.7. Retrograde Labeling of Rubrospinal Neurons

Four animals from each group were reanaesthetized and 5 *μ*L of 10% tetramethylrhodamine dextran dye (FluoroRuby; Molecular Probes, Eugene, OR) in PBS was applied on both rubrospinal areas of the proximal stump after a complete transection of the SC below the site of contusion at T12 in order to evaluate the survival of rubrospinal neurons. Five days later, the rats were decapitated and their brains were excised, processed, and cryosectioned. Every other 20 *μ*m thick section of the red nuclei (an average of 44 sections) was qualitatively and quantitatively examined by fluorescence and confocal microscopy. Only large and well-stained cells (with the whole body labeled) were counted. The total number of labeled cells was counted in every section from each brain. Thus, the number of labeled cells recorded for each brain is the sum of all the cells counted in each section. The average number of cells counted in its two red nuclei gives the number of labeled neurons in each rat. 

### 2.8. Number of Surviving Ventral Horn Neurons

Another four rats from each group were anaesthetized and perfused intra-aortically with 100 mL of PBS, pH 7.4 plus heparin (1%) at 4°C, followed by 400 mL of a fixative solution (4% paraformaldehyde in PBS, pH 7.4 at 4°C). One centimeter of tissue at the lesion site of spinal cord (0.5 cm caudal/rostral) was removed and incubated for 2 h in the same fixative solution and then cryoprotected in a 30% sucrose solution for at least 3 days. Afterwards, 3 sequential cryosections 10 *μ*m thick were cut at 0, 0.5, 1, 2, 3 and 4 mm caudal to rostral from the epicenter of injury. Hematoxylin and eosin-stained sections were analyzed for surviving ventral horn neurons. The number of surviving ventral horn neurons was confirmed and counted by the presence of Nissl substance, a euchromatic nucleus, and a nucleolus [24]. The average number of cells counted in three sequential sections gives the number of neurons in each rat. 

### 2.9. Griess's Method for Nitrites Determination

Approximately 500 *μ*L of whole blood was obtained from each animal and collected in heparin-containing tubes, which were then centrifuged for 5 min. at 300 rpm in order to collect 100 *μ*L of plasma. A total of 300 *μ*L of distilled water and 20 *μ*L of 30% zinc sulphate (ZnSO_4_) (Sigma-Aldrich, St. Louis, Mo, USA) were added before mixing and centrifuging the samples for 5 min. at 10,000 rpm to obtain the supernatant. 

A different solution was prepared by adding 0.5 g of cadmium to 2 mL of an aqueous 5% cupric sulphate (CuSO_4_) solution and mixed for 10 min. The solution was centrifuged and the supernatant was discarded, the pellet was washed three times: once with distilled water, once with hydrochloric acid (HCl) 0.1 N, and finally with 5% ammonium chloride (NH_4_Cl) pH 9. NH_4_Cl, which was later decanted from the tube and the sample plasma was added. The tubes were horizontally agitated for 15 min. and then centrifuged for 5 min. at 3500 rpm to take 200 *μ*L of the supernatant. This volume was supplemented with 700 *μ*L of distilled water and 50 *μ*L of a sulfanilamide solution at a concentration of 0.5 gr per milliliter of 15% acetic acid (Merck KGaA, Germany) and was incubated for 10 min. at room temperature (RT). Fifty microliters of N-(1-naphtyl)-ethylenediamine dichlorohydrate at a concentration of 0.2 g per milliliter of 15% acetic acid (Sigma-Aldrich, St. Louis, Mo, USA) were mixed with the samples and incubated for 30 min. at RT. Nitrite concentration was determined using a sodium nitrite (NaNO_2_) standard-curve and the absorbance was read at 540 nm (Jenway 6305 uv/vis). 

### 2.10. Thiobarbituric Acid-Based Determination of Lipid Peroxidation

Oxidative damage of lipids was determined through spectrophotometric quantification of the stable, chromogenic, and fluorescent adducts formed by the reaction of malondialdehyde (MDA) with 2-thiobarbituric acid (TBA).

A total of 100 *μ*L of plasma was obtained using the procedure described above, with the addition of 10 *μ*L of butirylhydroxytoluen (BHT) 2 mM. The resulting solution was diluted 1 : 5 with PBS and 400 *μ*L were taken in order to add 50 *μ*L of BHT 12.6 mM plus 400 *μ*L of ortho-phosphoric acid 0.2 M. This solution was mixed for 10 s and then 50 *μ*L of TBA 0.11 M were added and mixed again. Sample tubes were capped and incubated for 15 min. at 90°C in a double boiler water bath, they were cooled down on ice and then 1 mL of N-butanol and 100 *μ*L of a NaCl saturated solution were added. The tubes were vigorously shaken for 30 s and then centrifuged for 1 min. at 500 rpm. Afterwards, 500 *μ*L of the butanol phase was read at 535 and 572 nm in order to correct the absorbance. Estimates were done taking the MDA standard-curve as a reference.

### 2.11. iNOS, IL-10, and TGF-*β* Semiquantitative Expression

Gene expression of iNOS, IL-10, TGF-*β*, and HPRT (hypoxanthine phosphoribosyl transferase; housekeeping gene) [26] was determined through the quantification of mRNA transcripts performing real time PCR at 3 h, 7, and 14 days after injury. Total RNA was isolated from a 1.0 cm-long sample taken from the injury site on the spinal cord (0.5 cm caudal/rostral) using the Trizol method (Invitrogen, Carlsbad, CA, USA). cDNA was synthesized from 2 mg of total RNA using the Superscript II transcriptase enzyme and Oligo dT (Invitrogen, Carlsbad, CA, USA). The forward (F) and reverse (R) primers, amplicon size, and Gene Bank entry numbers were

 iNOS: (D)AAGCTGGTGGCCGCCAAGCT/(I)ATGTGAGGGGTTTGGGGGGA/258 pb/AY211532.1,
 IL-10: (D)GGGGTGACAATAACTGCA/(I)GGGGCATCACTTCTACCA/216 pb/NM_012854,
 TGF-*β*: (D)CCCAACCCCAGCTCCAAGCG/(I)CAGCCACTCTGCGGTGCCTC/132 pb/NM_013174.1,
 HPRT: (D)AAGCTTGCTGGTGAAAAGGA/(I)CAAAGCCTAAAAGACAGCGG/192 pb/NM_012583.2.

Reactions were performed in a final volume of 10 *μ*L according to supplier's recommendations using the Light Cycler FastStart DNA Master^PLUS^ SYBR Green I kit (Roche, Diagnostics, Indianapolis, USA) with 2 *μ*L of cDNA and 5–10 pM of each primer. Amplification was detected in a LigthCycler 2.0 (Roche Diagnostics, Indianapolis, USA) measuring the fluorescence of the SYBR Green incorporated during the reaction. PCR conditions were as follows: one cycle of denaturalization at 95°C/10 min. followed by 40 cycles of denaturalization at 95°C/10 s, aligning at 60°C/10 s, extension at 72°C/7 s, a dissociation curve at 65°C/1 min., and finally a cooling cycle at 40°C/30 s.

LightCycler 4.0 software was used (Roche Molecular Biochemicals) for the analysis of the amplification curves. PCR product identification was confirmed with the analysis of the dissociation curves. The reactions from each sample were carried out in duplicates using a reaction with no cDNA as control. Relative concentrations were calculated through the Ct method (i.e., the cycle number in which templates' exponential amplification begins) using the second derivative. An average of the values from each sample was obtained, as well as an average expression value from each analyzed gene, which was compared to that of the housekeeping gene, assigning a value equal to one as expression normalization.

### 2.12. Statistical Analysis

Data are expressed as mean ± standard error of the mean (SEM). Results were analyzed using a two-way ANOVA for repeated measures in the motor recovery experiment [27], and the nonparametric Mann-Whitney *U* test for: (a) survival of ventral horn and rubrospinal neurons and (b) relative expression of genes. Finally, a Kruskal-Wallis test was used for nitrite and MDA determinations. *P* values less than 0.05 were considered statistically significant.

## 3. Results

### 3.1. Motor Recovery Evaluations (Basso, Beattie, and Bresnahan Locomotor Ability Open-Field Test)

**Figure 1:**
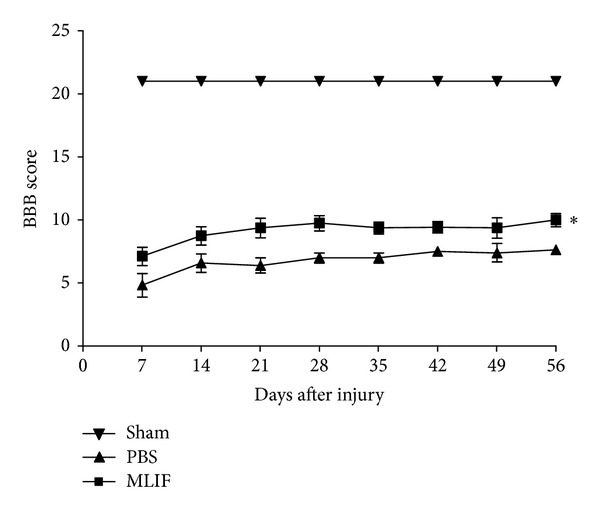
Motor recovery of rats subjected to SC injury. Rats were treated with MLIF (■) or PBS (▲). Motor recovery was assessed according to the locomotor ability open-field test scale. Evaluations took place once a week for 56 days. A significantly better motor recovery was observed in the group that received MLIF compared to the one that received PBS. *Different from PBS-treated rats (*P* = 0.03; two-way ANOVA for repeated measures). Each point represents the mean ± SEM of 10 rats. BBB: Basso, Beattie, and Bresnahan locomotor ability open-field test.

For the first part of this work we investigated the effect of MLIF on the motor recovery of animals subjected to a moderate SC contusion.  Figure 1 shows that animals receiving MLIF presented a better motor recovery achieving a higher score on the locomotor ability open-field test (9.14 ± 0.8, mean ± SEM; at day 56), compared with the animals treated with PBS (6.80 ± 0.9; at day 56). The difference was statistically significant (*P* = 0.03, two-way ANOVA for repeated measures). Sham animals presented the maximum value on locomotor ability open-field test scale. 

### 3.2. Survival of Rubrospinal Neurons

**Figure 2:**
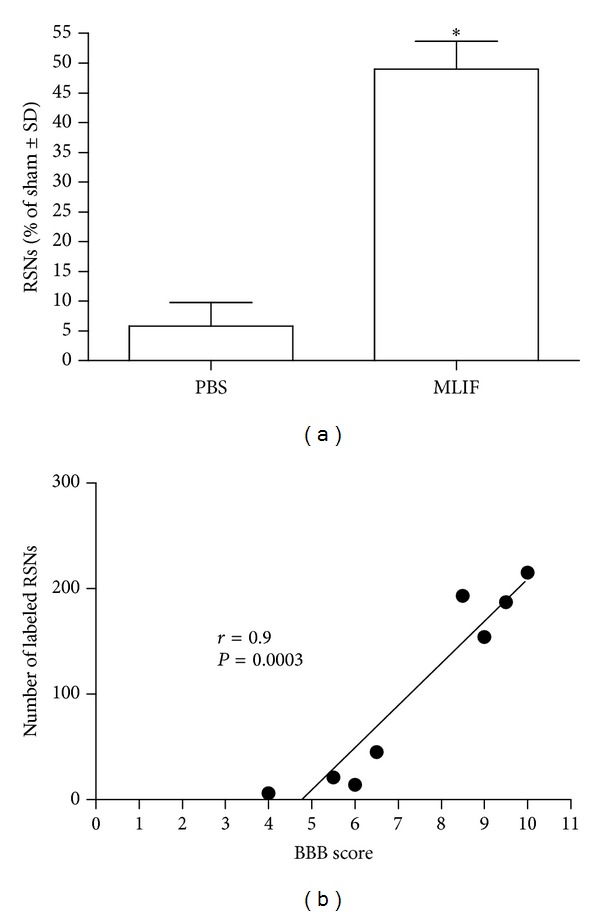
Neuronal survival in the red nucleus of rats with spinal cord injury treated with MLIF or PBS. The beneficial effect induced by MLIF therapy promoted a better survival of rubrospinal neurons (a). The number of surviving rubrospinal neurons correlated with the final motor recovery scores (b). *Different from PBS (*P* = 0.002, Mann-Whitney *U* test). Bars represent the mean ± SEM of 4 rats. BBB means: Basso, Beattie, and Bresnahan locomotor ability open-field test and RSNs: rubrospinal neurons.

Figure 2 depicts the survival of rubrospinal neurons. The group treated with MLIF showed a higher survival of rubrospinal neurons (185.3 ± 30%, from Sham animals: 49.1 ± 8, mean ± SEM), as compared to PBS-treated rats (21.3 ± 15%; 5.8 ± 3; *P* = 0.002, Mann-Whitney *U* test, Figure 2(a)). The total number of labeled red nuclei cells correlated with final motor recovery scores (*r* = 0.90, *P* = 0.0003) (Figure 2(b)).

### 3.3. Survival of Ventral Horn Neurons

**Figure 3:**
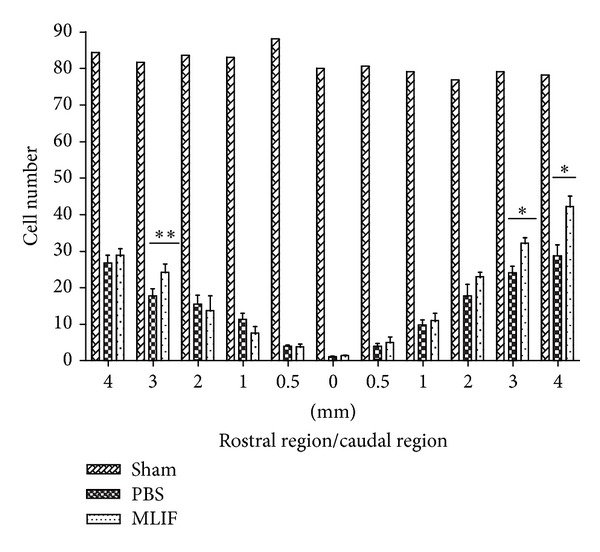
Survival of ventral horn neurons in rats with SC injury treated with MLIF or PBS. Ventral horn neurons in sham-operated animals are also depicted. MLIF treatment increased the number of ventral horn neurons at 3 mm rostral region and 3 and 4 mm caudal region the epicenter to the site of injury (*P < 0.001, Kruskal-Wallis followed by Mann-Whitney *U* test; **P < 0.05). Bars represent the mean ± SEM of 4 rats.

Figure 3 shows that MLIF increases the number of preserved ventral horn neurons at the site of injury, particularly in the caudal region, when compared to animals treated with PBS. A significant increase was found in MLIF-treated rats at 3 mm rostral to the epicenter (*P* < 0.05, Kruskal-Wallis followed by Mann-Whitney *U* test). Likewise, there were more residual ventral horn neurons at 3 and 4 mm towards the caudal region of the injury (*P* < 0.001).

### 3.4. iNOS and Cytokine Gene Expression

**Figure 4:**
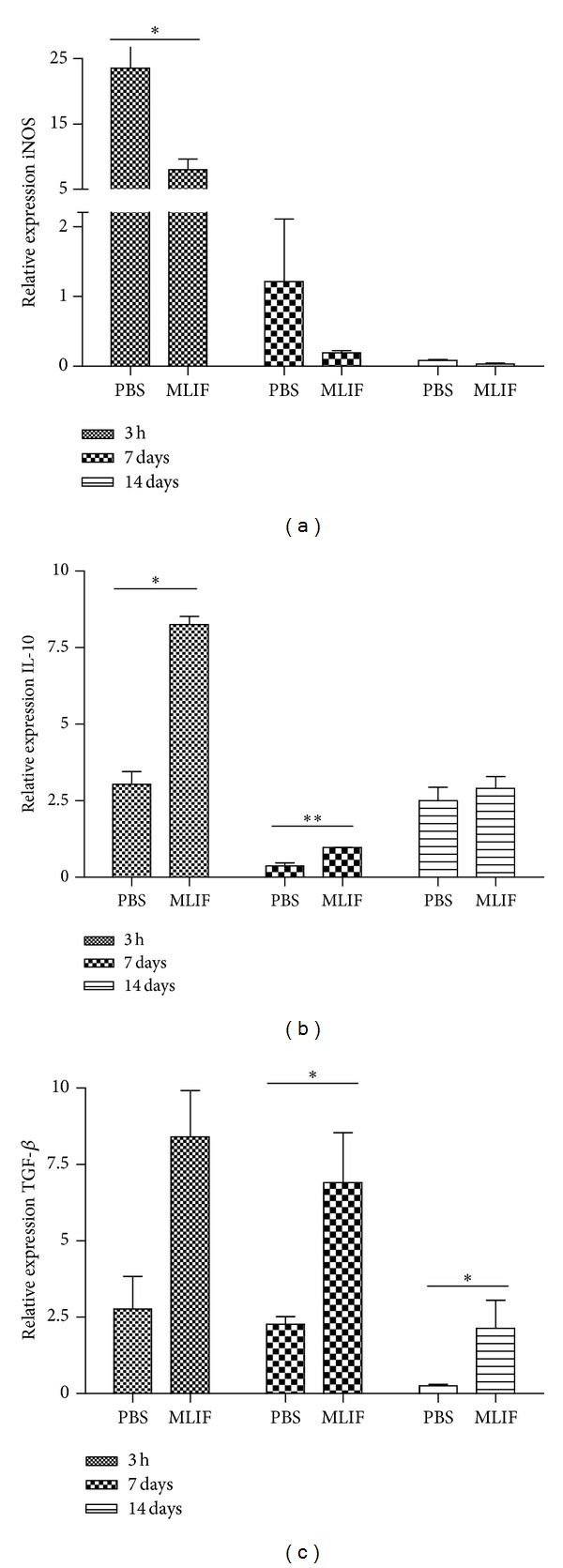
Relative expression of iNOS, IL-10, and TGF-*β*. The relative expression of genes was determined in the injury site of the spinal cord (0.5 cm caudal/rostral): (a) iNOS, (b) IL-10, and (c) TGF-*β* genes were analyzed through real-time PCR at 3 h, 7, and 14 days after SC injury in MLIF- or PBS-treated rats. MLIF reduced the relative expression of iNOS and increased IL-10 and TGF-*β* (*P < 0.05, Mann-Whitney *U* Test). Bars represent the mean ± SEM of 4 rats.

The effect of MLIF on the expression of anti-inflammatory genes in cell cultures led us to investigate whether the peptide is able to modify the expression of any of these genes (IL-10 and TGF-*β*) in vivo. Aside from this, we also explored the effect of MLIF on the expression of iNOS.  Figure 4 shows the relative expression of the genes encoding for iNOS, IL-10, and TGF-*β* (Figures 4(a), 4(b), and 4(c), resp.) at 3 h, 7, and 14 days after SC injury in rats treated with MLIF or PBS. The gene expression of sham-operated animals was used as a control and as a normalization point (assigning a value equal to 1).  Figure 4(a) shows that MLIF reduced the relative expression of iNOS at 3 h (*P* < 0.05) when compared to the PBS-treated group; at 7 and 14 days there was no significant difference. The relative expression of IL-10 was significantly increased at 3 h and 7 days after injury (Figure 4(b)). On the other hand, MLIF provoked an increase of TGF-*β* at all evaluated times (Figure 4(c)). There was a significant difference at 7 and 14 days after injury (*P* < 0.05).

### 3.5. Nitric Oxide Determination

**Figure 5:**
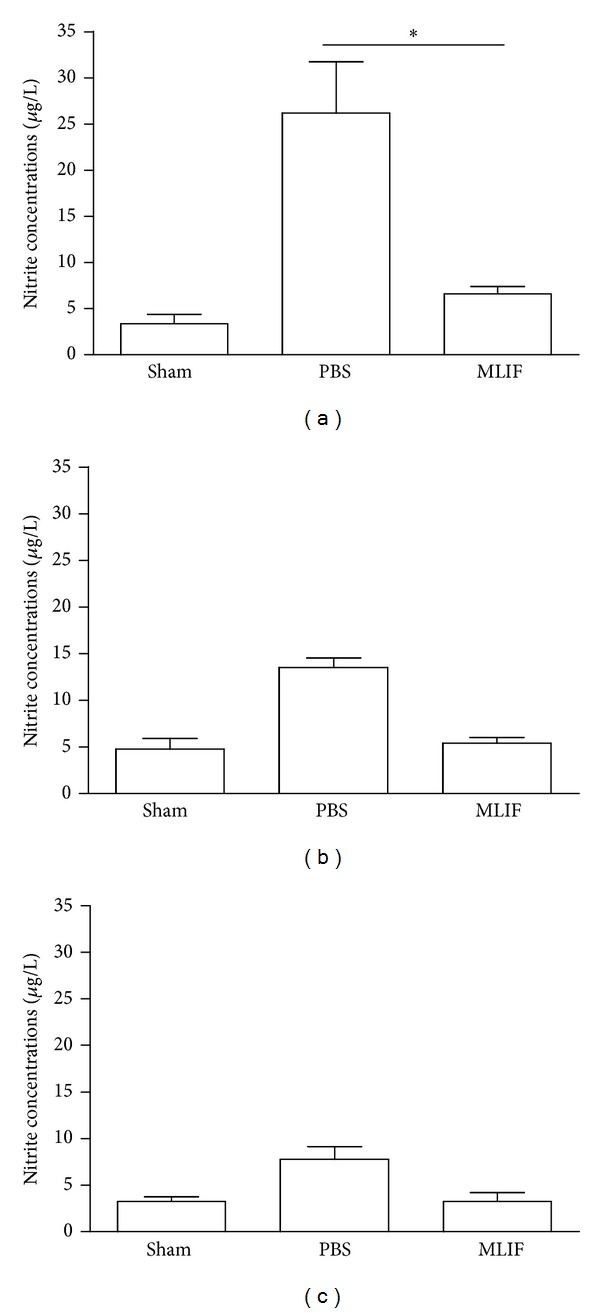
Nitrite determination in sham-operated and SC-injured rats. Nitrite concentration was assessed in plasma at 3 h (a), 7 (b), and 14 days (c) after injury in MLIF- or PBS-treated rats. MLIF decreased nitrite concentrations for all evaluated times, but only at 3 h was it significantly different to injured controls (*P < 0.05, Kruskal-Wallis test). The bars represent mean ± SEM of 4 rats.

Nitric oxide was determined detecting the production of nitrites in plasma at 3 h (Figure 5(a)), 7 days (Figure 5(b)), and 14 days (Figure 5(c)) after injury. Nitrite production decreased at all evaluated time points in rats treated with MLIF when compared to animals treated with PBS. Nevertheless there was only a significant difference (*P* < 0.05) at 3 h. Noteworthy, when comparing the MLIF-treated group to the sham-operated rats, there was no significant difference for any of the evaluated times (*P* > 0.05).

### 3.6. Lipid Peroxidation

**Figure 6:**
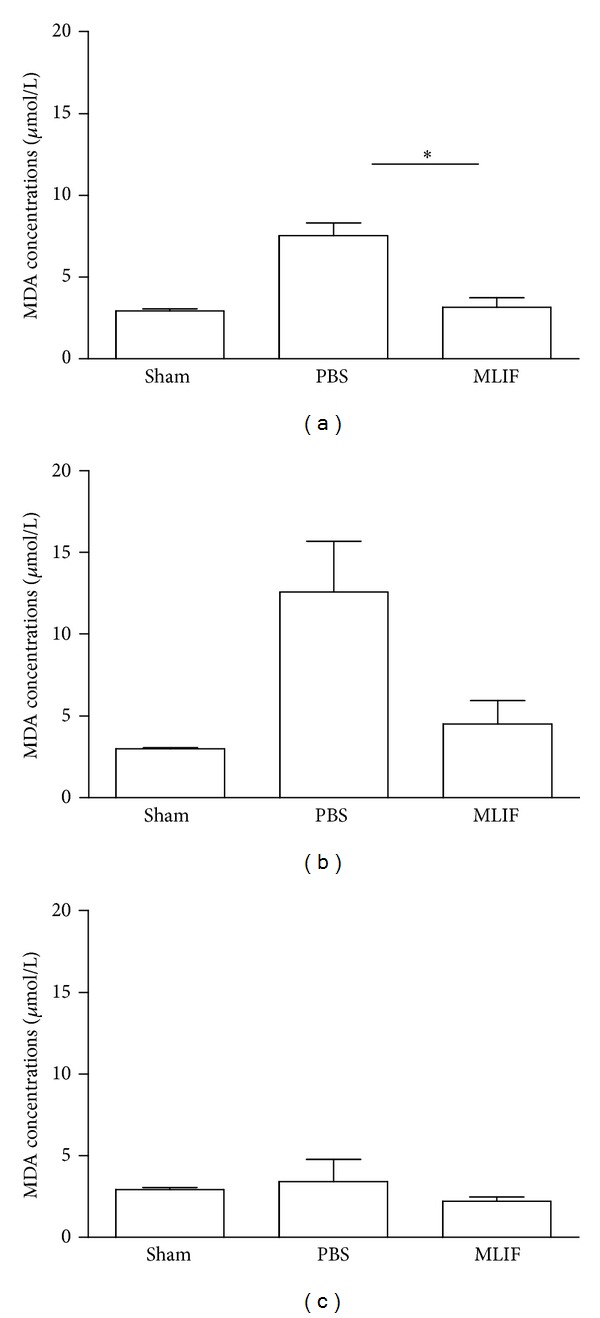
Lipid peroxidation in sham operated and SC injured rats. Lipid peroxidation was quantified in plasma at 3 h (a) 7 (b) and 14 days (c) after SC injury. MLIF reduced lipid peroxidation at all evaluated time points, however, the difference between MLIF- and PBS-treated rats was only statistically significant 3 h after injury (*P < 0.05; Kruskal-Wallis test). The bars represent mean ± SEM of 4 rats.

Three hours after injury, MLIF significantly reduced lipid peroxidation compared to PBS-treated rats (*P* < 0.05, Figure 6(a)). Although a reduction in MDA levels was observed, this was not significant at 7 (Figure 6(b)) or at 14 days (Figure 6(c)) after injury. Lipid peroxidation was similar between the sham-operated and SC-injured rats treated with MLIF.

## 4. Discussion

The pathophysiology of SC injury is characterized by the initial primary injury that is followed by secondary mechanisms of damage. The latter involve cascades of biochemical, molecular, and cellular changes, which can produce even more extensive damage. The inflammatory response and the consequent release of free radicals play a pivotal role in the degeneration of the SC tissue [27, 28]. A number of therapies that prevent oxidative and inflammatory processes have been proposed to avoid neurodegeneration and promote neuroprotection [29].

MLIF is an anti-inflammatory oligopeptide produced by *Entamoeba histolytica *[15] that has been shown to be capable of modulating the production of NO, ROI, proinflammatory cytokines, and the expression of genes related to axonal guidance and inflammation in vitro [17, 18]. These findings led us to investigate whether this peptide is capable of modifying some of the specific biological effects observed in vitrobut in an in vivo model of SC injury.

The most important parameter needed to evaluate a potential therapy for SC injury is functional recovery. An improvement in this area would translate into a clinically relevant treatment alternative; therefore, the first goal of this work was to evaluate the effect of MLIF on locomotor recovery of rats subjected to a moderate SC contusion. We demonstrated that treatment with MLIF significantly improved functional recovery when compared with a PBS control group.

A better functional status after SC injury suggests that more neurons survived the initial insult. To evaluate neuroprotection, we used the survival of two populations of neurons that are responsible for SC-mediated motor control. The number of surviving ventral horn neurons and the neurons of the rubrospinal tract allow us to evaluate the effectiveness of the protective microenvironment provided by MLIF [30]. Our study demonstrated that there were more functional axons within the rubrospinal tract of animals treated with MLIF. The integrity of the rubrospinal tract means that more electrochemical signals are able to travel from the red nucleus in the brain to the different segments of the SC, a key component in voluntary muscle control. This neuroprotective effect was also observed when we quantified the number of surviving ventral horn neurons at different distances from the epicenter of the injured SC. To further strengthen our analysis, this increment in neuronal survival correlated with a higher score on the locomotor ability open-field test. The previous data shows that MLIF was capable of providing neuroprotection. MLIF developed a microenvironment conducive towards tissue preservation and translated into significant functional recovery. 

To further elucidate how MLIF is responsible for developing this neuroprotective microenvironment, we evaluated the expression of several molecules strongly implicated in the secondary phase of SC injury. Nitric oxide overproduction is linked to the formation of neurotoxic intermediaries, such as peroxynitrite, aggravating tissue destruction and inflammation. This molecule is synthesized by nitric oxide synthase (NOS) in its two constitutive and one inducible isoforms: neuronal (nNOS or NOS1; constitutive), inducible (iNOS or NOS2), and endothelial (eNOS or NOS3; constitutive). After SC injury, the isoform responsible for the uncontrolled synthesis of NO is iNOS, which is activated in circulating immune cells as a response to CNS injury [31]. We demonstrated that iNOS gene expression was significantly reduced at 3 hours after SC injury in animals treated with MLIF. 

MLIF has demonstrated to inhibit monocyte locomotion and the synthesis of ROI in monocytes and neutrophils [18]. It has also been shown that MLIF increases the expression of neuroprotective cytokines such as IL-4, IL-10, and TGF-*β* [32]. We, therefore, decided to evaluate the expression of IL-10 and TGF-*β* to see if they were responsible for the increased neuroprotection induced by MLIF. Our study demonstrated that IL-10, a potent anti-inflammatory cytokine that suppresses the majority of microglial/macrophage responses, is in fact upregulated at 3 hours and even up to 7 days after treatment with MLIF [33]. The response observed for TGF-*β*, another anti-inflammatory cytokine with neuroprotective effects, had increased expression at 7 days and up to 14 days after injury. The effect seen for TGF-*β* is triggered later and lasts even longer than IL-10 expression. 

These results provide insight into the mechanisms of action of MLIF. We observed that most of these processes appear early in SC injury pathophysiology (i.e., before 3 hours). However, MLIF is a pentapeptide that when administered has a half-life of less than one minute. The reason for its short action time is that it is particularly vulnerable to cleavage by peptidases [34]. This poses an issue when trying to ascertain the downstream role of this immunomodulatory peptide. All the known effects for MLIF relate to immune cell targets (i.e., locomotion in monocytes, ROI synthesis in neutrophils) but very little is known on its effect in the CNS. Interestingly however, the first immune cell to arrive at the injured SC is the neutrophil, and this is approximately 3 hours later [35]. This means that some other cell is exerting MLIF's beneficial effects as these processes are taking place before neutrophilic extravasation. The direct effects of MLIF on neural tissue and CNS cells must further be studied. Nonetheless, this study has demonstrated that MLIF downregulates iNOS and upregulates IL-10 and TGF-*β* in the injured SC resulting in greater neuronal survival. The cell type responsible for the change in these gene expression patterns must also be investigated.

MLIF has also been successfully implemented as a therapy in acute ischemic stroke [36, 37]. A comprehensive study by Zhang et al. demonstrated that MLIF exerted its effect by binding to ribosomal protein translation elongation factor (eEF1A1). MLIF binds to domain 1 of eEF1A1, inhibiting the expression of pathological inflammatory adhesion molecules in endothelial cells of CNS blood vessels. These adhesion molecules are intercellular adhesion molecule 1 (ICAM-1) and vascular cell adhesion molecule 1 (VCAM-1). Another study by Gimenez-Scherer et al. has also demonstrated MLIF's capacity to decrease the expression of VCAM-1 in vascular endothelium [38]. The direct application of MLIF after injury allows for it to come into contact with the endothelial cells of the microvasculature of the SC. Circulating neutrophils are an important source of iNOS expression, NO production, and consequent lipid peroxidation. After CNS trauma, neutrophils increase 3- to 10-fold from 2 to 24 hours after SC injury [35]. This is secondary to the systemic inflammatory response observed after injury to the CNS. These circulating neutrophils also display a stronger oxidative burst, increased production of ROI, more myeloperoxidase, and upregulated iNOS and COX2 expression [39]. Neutrophils are covered with integrin CD11d/CD18, a molecule that interacts with ICAM-1 and VCAM-1 in order for leukocyte extravasation to take place [40]. However, the inhibition of adhesion molecule expression by MLIF would result in less neutrophilic activation and extravasation into the injured SC. Studies that have used monoclonal antibodies against CD11d/CD18 have obtained similar results to the ones seen in treatment with MLIF [39–41]. 

In order to measure the activation of the systemic inflammatory response and in an effort to correlate this with the secondary phase of injury, we decided to quantify the circulating NO levels and lipid peroxidation by-products in whole blood. Our results showed that MLIF administration significantly lowered systemic NO and lipid peroxidation at the 3-hour interval after SC injury. The exact relationship between plasma concentrations of nitrites and MDA/TBA and the actual intraspinal values of NO and lipid peroxidation must be further studied. The validation of these parameters would be useful in a clinical setting to track the intensity of the secondary phase of SC injury from an easily obtainable source (whole blood). 

The benefits of less activated neutrophils would take place prior to the 3-hour mark and therefore they are in line with the time frame observed in our study. It is likely that a reduction in activated neutrophils, as a consequence of the inhibition of adhesion molecule expression by MLIF, could contribute in the reduction of the circulating levels of NO and lipid peroxidation. We have successfully demonstrated that treatment with a pentapeptide synthesized by the parasitic protozoa *E. histolytica *is effective in the treatment of SC injury.

## 5. Conclusion

Treatment with intraspinal MLIF after SC injury causes downregulation of the iNOS gene expression which correlates with a lower systemic production of NO and lipid peroxidation. MLIF also increased the expression of neuroprotective anti-inflammatory cytokines such as IL-10 and TGF-*β*. All these resulted in greater preservation of ventral horn and rubrospinal tract neurons. More importantly, MLIF-based therapy yielded a significant improvement in neurological deficits and functional recovery. This work is the first to demonstrate the novel use of MLIF as a potential treatment for SC injury.
